# Markov Stability partitioning shows spectrally dependent community structure amongst thalamocortical neural ensembles

**DOI:** 10.1186/1471-2202-16-S1-P222

**Published:** 2015-12-18

**Authors:** Christian-David Martin, Silvia Ardila-Jimenez, Simon Schultz

**Affiliations:** 1 Centre for Neurotechnology & Department of Bioengineering, Imperial College London, London, UK

## 

**Figure 1:**
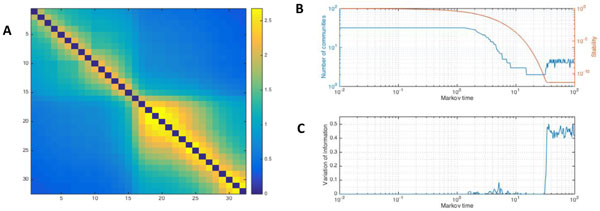
A. Mutual Information (MI) between the LFP time-series of each of 32 electrodes within mouse LGN. MI is computed with entropy estimates from k-nearest neighbor distances [2]. The diagonal was excluded from analysis. Two broad groupings can be discerned in this example. B. Application of the Markov Stability algorithm [3] to the time-series data in A. The number of communities detected is depicted as a function of Markov time (blue), with plateau phases indicative of stable partitioning. Stability (orange) of a given partition is defined as the probability that a random walker at stationarity starts in community i and ends up in the same community after time tM , minus the probability of this happening by chance, summed over all communities and nodes. C. Variation of information (VI), an information-theoretic measure of the distance between partitions, averaged over multiple runs of the algorithm (>100x). Low values are indicative of reliable partitioning. Partitioning into two and three groups is relatively stable across Markov time, and coincides with low VI, thus reflecting a meaningful partitioning.

The processing of information through the spatiotemporal coordination of neuronal activity is still poorly understood [1]. Here we analyse local field potential (LFP) signals from multi-electrode recordings in the mouse lateral geniculate nucleus (LGN) and visual cortex (V1), to systematically investigate interactions between neuronal ensembles across the frequency spectrum. Computing mutual information for each pair of electrodes using the k-nearest neighbor method developed by [2], two broad groupings can be discerned among the electrodes (Figure 1A). The same partitioning is found as a stable solution when applying the Markov Stability algorithm developed by Billeh et. al. [3], which uses a Markov diffusion process through the dataset to detect stable groupings (Figure 1B/C). Analysing narrowband filtered LFP signals, neuronal groupings were found to change between low (1-40 Hz) and high (>40Hz) frequency bands. One particular neural ensemble was found to participate in different groupings across low and high frequency bands, with differing interaction partners and mechanisms as assessed by phase-phase and phase-amplitude correlation measures, both within and across areas. This frequency-specific interaction pattern may allow for the simultaneous coordination of information transmission across different timescales.
